# Supplementary material for: Spatial analysis of mortality due to congenital syphilis in Brazil from 2008 to 2022
Source: PLoS One. 2026 Feb 3;21(2):e0326407. doi: 10.1371/journal.pone.0326407 (PMC12867209; doi:10.1371/journal.pone.0326407)
Supplement: Attachment — Submitted filename: Response to reviews.docx [file pone.0326407.s003.docx]

**PONE-D-25-29476R1**

**RESPONSE LETTER TO REVIEWERS AND EDITORS**

Dear Editors and Reviewers,

We would like to express our sincere gratitude for the careful review and thoughtful comments provided on our manuscript entitled “**Spatial analysis of mortality due to congenital syphilis in Brazil from 2008 to 2022**”. The suggestions and observations have been thoroughly considered and have significantly contributed to the improvement of the manuscript. Below, we present our point-by-point responses, outlining the changes made to the text and providing justifications where necessary. Should there be any remaining questions or a need for further modifications beyond those already made, we remain at your disposal.

**Reviewer #1:**

**[1] Introduction**

1. The authors could better contextualize the scenario of syphilis in Brazil. I strongly recommend that the authors delve a little deeper into the history of syphilis in Brazil.

**Response: Adjusted. Paragraph 3 and 4 of the introduction.**

2. Overview of the epidemiological scenario regarding syphilis in the world. To do this, they can include data from the United States, Canada, China and countries in the European Union. This type of information is important because it shows that syphilis is not a problem only in developing or low- and middle-income countries.

**Response: Adjusted. Paragraph 1 of the introduction.**

**[2] Methodology**

1. The data can be made available in a file with the extension “.csv” as a supplementary file to the manuscript. DATASUS, PNUD, IBGE.

**Response: The database was made available in the supplementary files.**

**[3] Discussions**

1. The limitations of the study are not addressed.

**Response: Adjusted. Limitations were inserted at the end of the discussion.**

2. Despite having submitted a report on the elimination of vertical transmission of HIV to the World Health Organization (WHO), Brazil has indicators of congenital syphilis that point in the opposite direction. How is this possible if women notified of HIV during pregnancy are subject to the same health system as women notified of syphilis?

**Response: Adjusted. Paragraph 18 and 19 of the discussion.**

3. The indicators of congenital syphilis in Brazil are the same or worse when compared to countries in the sub-Saharan region of Africa. How is this possible? We know that Brazil has a much more developed primary health care system than countries in the sub-Saharan region of Africa. Another point that draws attention is that countries in the sub-Saharan region of Africa maintain poor indicators for HIV (vertical transmission) and for Syphilis (vertical transmission), and not only for syphilis, as seems to be the case in Brazil.

**Response: Adjusted. Paragraph 16 and 17 of the discussion.**

4. Is there not a problem in the definition of congenital syphilis cases in Brazil? Does Brazil actually investigate cases of congenital syphilis? Regarding notification of the cause of death, which Health Information System does Brazil use for this? Is it an easy-to-use system? Does it really ensure that deaths are recorded appropriately?

**Response: Adjusted. Paragraph 1 and 2 of the discussion.**

5. In Brazil, I remember a great debate about the notification of deaths during the COVID-19 pandemic, precisely because health professionals had difficulty using ICD-10 in the death notification system. Does Brazil have a system for monitoring cases of syphilis in pregnant women that allows public health authorities to assess these cases in real time (online) and monitor follow-up?

**Response:** **Adjusted. Paragraph 3 of the discussion.**

6. There are publications of articles by Brazilian researchers reporting problems in reporting cases of congenital syphilis. Brazil also appears to have published a report that addresses this same issue - more than 90% of reported cases of congenital syphilis are based on doubt. Why does this happen in Brazil?

**Response: We greatly appreciate your contributions to our study. They were certainly crucial in enriching it. Regarding the information that 90% of cases are reported based on doubt, could you send us this reference for analysis? However, we would like to share these studies in advance, which discuss the difficulty of diagnosing congenital syphilis with the tests available in the Brazilian system. We highlight an important excerpt on this topic:**

***“Most newborns (NB) are asymptomatic at birth and gold standard tests for diagnosis, such as dark field Treponema research and polymerase chain reaction (CRP), are not available in clinical practice. The detection of IgG antibodies against Treponema in newborns does not allow the diagnosis of congenital infection due to the maternal-fetal transfer of these antibodies, and it is not possible to distinguish maternal antibodies from endogenous antibodies produced by the offspring. The detection of specific IgM treponemal antibodies, which do not cross the placental barrier, would confirm fetal infection, however, these laboratory tests have low sensitivity, so they are not validated for commercial use”.***

Pascoal LB, et al. Maternal and perinatal risk factors associated with congenital syphilis. <https://onlinelibrary.wiley.com/doi/10.1111/tmi.13881>

Arrieta AC, et al. Congenital syphilis: Clinical manifestations, evaluation, and diagnosis. <https://www.uptodate.com/contents/congenital-syphilis-clinical-manifestations-evaluation-and-diagnosis>

**Reviewer 2:**

Reviewer #2: Dear authors,

Although the importance of the addressed subject, I made some suggestions to improve the quality of results and, consequently, of the manuscript. Please, see the suggestions bellow:

Introduction:

**Comment 1:** The authors should evidenced that the studied problem is not only restricted to Brazil. Nothing was told about the epidemiological scenery worldwide; Quite confuse these lines "Between 2011 and 2021, there was a 39.9% rise in deaths from the disease nationwide. Moreover, during the same period, the infant mortality rate due to CS increased by 84.6%, rising from 3.8 to 7.0 deaths per 100,000 live births [2]:" In the introduction sounds that Brazil doesn't have any policy to fight the addressed problem. I suggest the authors talked a little bit about it; What was the reason for choosing the studied period (2008-2022)? In the sentence "based on the most recent data provided by the Brazilian government over a broad historical period (2008 to 2022)—" sounded you had a reason for that.

*Response:* We appreciate the reviewer’s comment. We have carefully reviewed the points raised and made the necessary revisions which are highlighted in the manuscript. As requested, we have included global data on congenital syphilis mortality, emphasizing that this is a global health issue not restricted to Brazil (see first paragraph). In addition, we have rewritten the section the reviewer found unclear (see second paragraph). Finally, we have listed the main strategies and policies adopted in Brazil that are related to the reduction of congenital syphilis cases and deaths (see third paragraph). Regarding the justification, the time frame was defined based on data availability. However, we agree with the reviewer and have now included this justification in the Methods section.

Methods:

**Comment 2:** I suggest the authors explained a little bit more about the study scenery. It is very well known the social inequity among the Brazilian regions. What about prenatal coverage? What about medical coverage? What about geography discrepancies?

*Response:* We have included information regarding the geographic division of Brazil, as well as key indicators to contextualize the territory, such as population size, land area, Human Development Index (HDI), and primary health care coverage rate in the country (see second paragraph of the Methods section).

**Comment 3:** There should be a map of Brazil showing the states and region limits to facilitate the reading and comprehension;

*Response:* Done. Thanks for the recommendation.

**Comment 4:** Nothing was told about the criteria for congenital syphilis (CS) diagnosis. Did you considered it when collected the data? This should be mentioned since you are working with the CS mortality rate;

*Response:* We have included, in the fifth paragraph of the Methods section, the criteria for the definition of congenital syphilis used in Brazil, which were consequently adopted for the present study.

**Comment 5:** Why did you consider such few independent variables? For example, you only focused on primary nurse and not medical doctors. CS is great influenced by health coverage, schooling level, the gestational age of the first antenatal care, socioeconomic conditions, number of prenatal consults, adequate treatment, .... I suggest you to expand your variables;

*Response:* A considerable number of studies have already examined the variables mentioned (Costa et al., 2024; Dalazen et al., 2022; Seabra et al., 2022; Dantas et al., 2023). Therefore, we decided to include variables that we consider important but that have been scarcely addressed in previous studies. This constitutes one of the novel aspects and strengths of the present study.

**Comment 6:** In autocorrelation analysis, considering the non-stationary of the data (please, apply statistic test to comprove it!), I suggest the authors to also apply GI statistics. Remember to define what you mean by group. For example, some studies consider a cluster only those composed by 3 or more municipalities. Additionally, you should apply False Discovery Ratio (FDR) test to exclude the non-significant clusters. Additonally, considering the temporal subject, I suggest the authors to employ the spatiotemporal risk analysis and not only focus on spatial risk.

*Response:* We thank the reviewer for the pertinent methodological suggestion. However, considering the objectives and design of the present study, we chose not to incorporate the Gi (Getis-Ord) statistic and the False Discovery Rate (FDR) test for the following reasons:

1. Analytical focus on local spatial autocorrelation: The objective of our study was to identify spatial autocorrelation patterns of congenital syphilis mortality in Brazil, using a classical approach for cluster detection through the Local Moran’s I (LISA) statistic. This choice is justified by the ability of Local Moran’s I to assess spatial relationships between municipalities based on similarity (like-with-like), which is particularly appropriate for data with heterogeneous national-scale distribution.
2. Complementarity (not mandatory use) of the Gi statistic: The Gi (Getis-Ord) statistic is indeed a useful tool to identify absolute concentrations of high or low rates (hotspots and coldspots), but it does not replace nor invalidate the use of Local Moran’s I. On the contrary, it constitutes an alternative or complementary approach that addresses a different analytical question—focused on extreme values rather than spatial similarity. Thus, joint application is recommended when the aim is to deepen the analysis from different perspectives, which was not the focus of the present study.
3. Methodological coherence and stability: The use of local empirical Bayesian smoothing combined with Local Moran’s I enabled us to reduce instability in municipalities with small counts and to identify spatial clusters in a statistically robust manner, as extensively validated in the epidemiological and geospatial literature.
4. Avoidance of methodological overfitting and multiple testing inflation: The application of the FDR test, while useful for controlling Type I errors in multiple testing scenarios, is not a standard methodological requirement in LISA analyses, especially when significance maps are already based on random permutations (as was the case in this study). This approach minimizes the likelihood of detecting spurious clusters. Foundational studies in the field (Anselin, 1995; Kulldorff et al., 2005; Waller & Gotway, 2004) also do not employ FDR as a mandatory procedure.
5. On non-stationarity: Indeed, the distribution of congenital syphilis mortality may exhibit spatial non-stationarity. However, Local Moran’s I already accounts for this variability by allowing for local (rather than global) analysis of spatial autocorrelation. The application of formal tests for spatial non-stationarity (such as Geographically Weighted Regression – GWR) is more appropriate in explanatory models, rather than in the exploratory framework proposed in this study.

In light of the above, we believe that the analytical approach adopted is methodologically consistent with the study’s objectives and aligned with best practices in spatial epidemiology. Nevertheless, we acknowledge that the incorporation of the Gi statistic, FDR tests, or more complex spatial modeling techniques may be appropriate in future investigations with a more explanatory analytical focus.

**Comment 9:** Again, GWR was very restricted to few idependent variables. Furthermote, nothing was told about the spatial dependency of OLS and GWR residuals. Just remembering that AIC is for OLS and AICc for GWR. What was the method you applied in OLS? Stepwise?

*Response:* We appreciate the observation, which is highly pertinent to the methodological refinement of the study. Indeed, the Geographically Weighted Regression (GWR) analysis was conducted using a limited number of independent variables, which we acknowledge as a limitation. This choice was deliberate, considering the multicollinearity identified among certain variables and the need to ensure the robustness of the local models, given GWR’s sensitivity to the number and correlation of predictors. We have reinforced this limitation in the revised version of the Discussion section (see the penultimate paragraph of the Discussion).

Regarding the Ordinary Least Squares (OLS) regression, the process was conducted as follows: initially, bivariate correlations between the independent variables and the congenital syphilis mortality rate were assessed. Variables showing a statistically significant association (p < 0.10) were considered candidates for inclusion in the multivariable model. Subsequently, a stepwise method was applied with entry and retention criteria of p < 0.10 and removal at p > 0.10. Multicollinearity was assessed using the Variance Inflation Factor (VIF), and only variables with VIF < 5 were retained in the model. Final model selection was based on the highest adjusted R² and the lowest Akaike Information Criterion (AIC) value, as recommended for global models.

Results:

**Comment 10:** I bet the results will change a lot after data reanalysis. Therefore, I will only evaluate in the second version. However, I suggest you to construct the figures separately for each topic. For example, Fig 1 should show the spatial distribution and autocorrelation; Fig 2 spatiotemporal and spatial risk analysisl, Fig. 3 GWR;

*Response:* We appreciate the reviewer’s insightful comment. We understand that the suggestion regarding the construction of figures separately for each topic has already been addressed in the current version of the manuscript. However, we remain open and willing to make further adjustments if the reviewer considers them necessary.

**Comment 11**: - Title of figures should express the intention of them. For example, Fig 2 show the beta coefficients? and 3 the local r2? Although we referred the figs in the manuscript, the titles were quite vagues.

*Response:* We thank the reviewer for this valuable suggestion. We have revised the titles of the figures to clearly express their purpose, making them more descriptive and aligned with the content presented.

**Comment 12:** Be careful when applying "," in numbers . In English we use "." ;

*Response:* Done. Thanks.

Discussion:

**Comment 13:** Discussion was focused exclusively in SDH, while policies were discarded. For example, the authors considered the low access to primary healthcare in North associated to CS mortality while in Rio is offered greater access. Previous studies in Rio de Janeiro showed that it is not true. The great problem in fighting CS in Rio is basically the unequal access mainly in women living in slams. The high-high cluster in North is composed by rich municipalities in Pará and they attracted immigrant searching for better life conditions. Also, the ones in Southeast have the greater primary healthcare coverage. Consider evaluate previous publications exploring the CS problem. The cluster in Rio is basically composed by the metropolitan municipalities. A super inequity region;

*Response:* We thank the reviewer for this important observation. We acknowledge that the discussion in the original manuscript focused primarily on social determinants of health (SDH) and that the role of health policies, particularly regarding primary healthcare access, deserves further attention. We have revised the Discussion section to better incorporate these aspects, including evidence on inequalities in access to care within regions such as Rio de Janeiro, as well as relevant literature addressing these complexities. We also highlighted the socioeconomic heterogeneity within clusters, as noted by the reviewer (see Discussion section).

**Comment 14:** Please, consider taking into account the efficiency of polices fighting CS in the address problem;

*Response:* Feito.

**Comment 15:** Discussion and data quality will increase after reanalyzing data;

*Response:* Thanks for the comment.

**Comment 16**: The study limit is already know for the ecologic study. I suggest to exclude it;

*Response:* Thanks for the comment.

**Comment 17:** This sentence "Finally, we recommend that further studies explore the reasons behind CS-related deaths in regions with better socioeconomic conditions, as these areas would ideally exhibit lower morbidity and mortality due to better access to healthcare services", should be moved to conclusion;

*Response:* Done. Thanks.

**Comment 18:** Additionally, conclusion should answer all the goals of the paper. Nothing was told about the GWR results…

*Response:* Done. Thanks.

We thank the reviewers once again for their careful evaluation and valuable comments, which have greatly contributed to improving our manuscript. We remain at your disposal for any further clarifications or additional revisions that may be required.

**REFERENCES**

Anselin, L. (1995). Local indicators of spatial association—LISA. *Geographical Analysis*, 27(2), 93–115.

Costa IB, Pimenta IDSF, Aiquoc KM, Oliveira ÂGRDC. Congenital syphilis, syphilis in pregnancy and prenatal care in Brazil: An ecological study. PLoS One. 2024 Jun 25;19(6):e0306120.

Dalazen CE, de Souza AS, Ribeiro CJN, Marques Dos Santos M, Probst LF, Theobald MR, De-Carli AD. Space-time risk cluster and time trends of congenital syphilis in Brazil: an ecological study. Trans R Soc Trop Med Hyg. 2022 Sep 10;116(9):822-831.

Dantas JDC, Marinho CDSR, Pinheiro YT, Ferreira MÂF, da Silva RAR. Temporal trend and factors associated with spatial distribution of congenital syphilis in Brazil: An ecological study. Front Pediatr. 2023 Mar 22;11:1109271.

Kulldorff, M. et al. (2005). A spatial scan statistic for retrospective surveillance. *Statistics in Medicine*, 24(24), 3959–3970.

Seabra I, Ferreira GRON, Sorensen W, Oliveira C, Parente AT, Gir E, Reis RKK, Ferrari RAP, Botelho E. Spatial scenery of congenital syphilis in Brazil between 2007 and 2018: an ecological study. BMJ Open. 2022 Apr 20;12(4):e058270.

Waller, L. A., & Gotway, C. A. (2004). *Applied Spatial Statistics for Public Health Data*. Wiley-Interscience.
