# Supplementary material for: Spatial analysis of mortality due to congenital syphilis in Brazil from 2008 to 2022
Source: PLoS One. 2026 Feb 3;21(2):e0326407. doi: 10.1371/journal.pone.0326407 (PMC12867209; doi:10.1371/journal.pone.0326407)
Supplement: Attachment — Submitted filename: Response to the Editor and Reviewers.docx [file pone.0326407.s004.docx]

**Response to the Editor and Reviewers**

**Manuscript title:** PONE-D-25-29476R1

**Manuscript ID:** SPATIAL ANALYSIS OF MORTALITY DUE TO CONGENITAL SYPHILIS IN BRAZIL FROM 2008 TO 2022

Dear Editor,

We would like to thank you and the reviewers for the valuable time and constructive comments provided to improve our manuscript. We carefully revised the paper in accordance with all suggestions. Below we present a detailed, point-by-point response describing how each comment was addressed.

All changes have been incorporated into the revised version of the manuscript, with corresponding modifications made in the *Methods*, *Results*, and *Discussion* sections.

**Response to the Editor**

**Editor’s comment:**

“The analyses related to the predictor variables must be revised. The justification presented for the non-inclusion of relevant variables is inadequate. Even if certain variables have been examined in prior studies, they must still be included to ensure appropriate control for confounding factors, allow collinearity assessment, and strengthen the contribution of your results to the literature. The very low R² values in your models reinforce the need to include a broader set of determinants. Furthermore, given the spatial nature of the data and the presence of spatial dependence, it is unlikely that the OLS model is the most appropriate. Therefore, the data should be reanalyzed with a broader set of explanatory variables and also considering additional models such as the Spatial Lag Model (SLM) and the Spatial Error Model (SEM), in addition to GWR.”

**Authors’ response:**

We thank the Editor for this constructive and technically relevant feedback, which led to substantial methodological improvements in the revised version of our study.

In response, we reanalyzed the data with an expanded set of explanatory variables, including socioeconomic indicators (Gini Index, Municipal Human Development Index), healthcare resources (density of physicians and primary care nurses), and prenatal care indicators (early initiation, ≥7 consultations, and treatment adequacy). These additions ensured appropriate control for confounding and allowed collinearity assessment (VIF < 5).

Considering the spatial dependence revealed by the LISA results and the significant Moran’s I of OLS residuals (I = 0.23; p < 0.001), we fitted two additional spatial econometric models — the **Spatial Lag Model (SLM)** and the **Spatial Error Model (SEM)** — using a queen contiguity matrix. The SLM yielded a significant spatial autoregressive parameter (ρ = 0.31; p < 0.001), while the SEM showed a significant spatial error term (λ = 0.28; p < 0.001), confirming spatial interaction and residual dependence. Both models improved fit relative to OLS (AIC = –8,875 for SLM; –8,879 for SEM), whereas the **GWR** remained the best-performing model (AICc = –8,880; R² = 0.56).

The revised manuscript now incorporates:

(i) a broader and conceptually grounded set of determinants;
(ii) complementary spatial models (OLS, SLM, SEM, and GWR); and
(iii) a strengthened discussion of the spatial and social determinants of congenital syphilis mortality in Brazil.

These refinements substantially improved the analytical rigor and external validity of the findings.

**Response to Reviewers**

**Reviewer 2**

**Comment 1:** “Therefore, the data should be reanalyzed with a broader set of explanatory variables and also considering additional models such as the Spatial Lag Model (SLM) and Spatial Error Model (SEM), in addition to GWR.”

**Response:** We thank the reviewer for this valuable methodological recommendation. We fully agree that incorporating additional spatial models can provide complementary insights into spatial dependence and the mechanisms underlying congenital syphilis (CS) mortality.

To address this, we conducted a reanalysis including a broader set of explanatory variables (physician density, PHC coverage, prenatal indicators, treatment adequacy). Moran’s I of the OLS residuals (I = 0.23; p < 0.001) confirmed spatial dependence, justifying the use of spatial autoregressive models.

We fitted both **SLM** and **SEM** using a queen contiguity matrix. Both yielded consistent results, with the same direction of associations observed in the GWR. The SLM presented ρ = 0.31 (*p* < 0.001), and the SEM showed λ = 0.28 (*p* < 0.001), confirming spatial interaction and residual dependence. Both improved model fit compared to OLS (AIC = –8,875 for SLM; –8,879 for SEM), while GWR remained superior (AICc = –8,880; R² = 0.56).

These analyses confirm the robustness of our results across different model specifications.

**Comment 2:** “Why did you consider so few independent variables? For example, you focused only on primary care nurses and not on physicians. Congenital syphilis (CS) is strongly influenced by health coverage, education, gestational age at first prenatal visit, socioeconomic conditions, number of prenatal consultations, and adequate treatment, among others. I suggest that you expand your variables.”

**Response:** We sincerely thank the reviewer for this important comment. In the revised version, we expanded the analytical model to include:

- physician density per 10,000 inhabitants;
- PHC coverage;
- proportion of women initiating prenatal care ≤12 weeks;
- proportion of women with ≥7 prenatal visits;
- proportion of adequately treated pregnant women;
- MHDI; and
- Gini Index.

All variables were standardized and tested for collinearity (mean VIF = 2.8). The expanded model improved explanatory power (OLS: R² = 0.50; GWR: R² = 0.56) and retained theoretically consistent associations — inequality positively associated with mortality, and healthcare access and quality inversely associated. These revisions are reflected in the *Methods*, *Results (Table 2)*, and *Discussion* sections.

**Comment 3:** “In autocorrelation analysis, considering the non-stationarity of the data (please, apply a statistical test to prove it!), I suggest the authors also apply Gi statistics. Remember to define what you mean by group... Additionally, you should apply a False Discovery Ratio (FDR) test... I also suggest employing a spatiotemporal risk analysis.”

**Response:** We appreciate this detailed methodological recommendation. In response:

1. **Non-stationarity:** The **Koenker (Breusch–Pagan)** test confirmed spatial non-stationarity (*p* < 0.001).
2. **Local clustering:** The **Getis–Ord Gi*** analysis identified *hot* and *cold spots*; clusters were defined as ≥3 contiguous municipalities.
3. **Multiple testing:** The **FDR correction** (Benjamini–Hochberg) was applied (*q* < 0.05), retaining only significant clusters.
4. **Spatiotemporal analysis:** We applied the **space–time scan statistic (SaTScan)** with a discrete Poisson model (2008–2022), identifying persistent and emerging clusters.

These steps enhanced the spatial and temporal robustness of the analysis. Updates appear in *Methods* and *Results*.

**Comment 4:** “Again, GWR was very restricted to few independent variables. Furthermore, nothing was told about the spatial dependency of OLS and GWR residuals. Just remembering that AIC is for OLS and AICc for GWR. What was the method you applied in OLS? Stepwise?”

**Response:** We refined model specification and diagnostics as follows:

1. **Variable selection:** The final GWR included Gini Index, MHDI, nurse density, non-treponemal tests, and physician density, based on theoretical relevance and VIF < 5.
2. **OLS estimation:** The OLS used **backward stepwise selection** guided by AIC and significance (*p* < 0.05).
3. **Residual dependency:** Moran’s I of residuals was significant for OLS (I = 0.23; *p* < 0.001) but non-significant for GWR (I = 0.04; *p* = 0.210).
4. **Model fit:** Fit indices were corrected (AIC for OLS; AICc for GWR): AIC = –8,870; AICc = –8,880.

These refinements enhanced model transparency and were incorporated into *Methods* and *Results*.

**References**

Anselin, L. *Spatial Econometrics: Methods and Models.* Springer, 1988.

Fotheringham, A.S., Brunsdon, C., & Charlton, M. *Geographically Weighted Regression: The Analysis of Spatially Varying Relationships.* Wiley, 2002.

**Sincerely,**

Yago Tavares Pinheiro, PhD
