# Supplementary material for: Spatial analysis of mortality due to congenital syphilis in Brazil from 2008 to 2022
Source: PLoS One. 2026 Feb 3;21(2):e0326407. doi: 10.1371/journal.pone.0326407 (PMC12867209; doi:10.1371/journal.pone.0326407)
Supplement: Attachment — Submitted filename: Response to Reviewers (4).docx [file pone.0326407.s005.docx]

**Response to Reviewers**

**Manuscript title:** PONE-D-25-29476R1

**Manuscript ID:** SPATIAL ANALYSIS OF MORTALITY DUE TO CONGENITAL SYPHILIS IN BRAZIL FROM 2008 TO 2022

Dear Editor,

We would like to thank you and the reviewers for the valuable time and constructive comments provided to improve our manuscript. We carefully revised the paper in accordance with all suggestions. Below we present a detailed, point-by-point response describing how each comment was addressed.

All changes have been incorporated into the revised version of the manuscript, with corresponding modifications made in the *Results* section.

**Response to Reviewers**

**Reviewer**

**Comment 1:** “While you have made clear improvements in the manuscript, including the incorporation of the SLM and SEM models, only the AIC values and spatial parameters (ρ and λ) are presented, while the regression coefficients for the explanatory variables in these models are missing. Please include a concise table reporting the coefficients (β) and p-values for the SLM and SEM models. This will ensure transparency and allow readers to confirm whether the associations remain consistent across models. The Discussion should also better articulate and compare the results obtained from the three modeling approaches (OLS, SLM/SEM, and GWR).”

**Response:** The reviewer's suggestion was accepted, and the information was added to Table 3 (Results section). Additionally, a brief discussion of the new results was included in the third paragraph of the Discussion section.

**Sincerely,**

Yago Tavares Pinheiro, PhD
